# Pharmacological Treatment of Patients with Metastatic, Recurrent or Persistent Cervical Cancer Not Amenable by Surgery or Radiotherapy: State of Art and Perspectives of Clinical Research

**DOI:** 10.3390/cancers12092678

**Published:** 2020-09-19

**Authors:** Angiolo Gadducci, Stefania Cosio

**Affiliations:** Department of Clinical and Experimental Medicine, Division of Gynecology and Obstetrics, University of Pisa, 56127 Pisa, Italy; stefania.cosio@gmail.com

**Keywords:** cervical cancer, chemotherapy, cisplatin, paclitaxel, bevacizumab, immune checkpoint inhibitors, phosphatidylinositol 3-kinase (PI3K) inhibitors, PARP inhibitors

## Abstract

**Simple Summary:**

The aim of this review is to assess the available literature of the pharmacological treatment of patients with metastatic, recurrent or persistent cervical cancer not amenable by surgery or radiotherapy. The results and toxicities of cisplatin-based doublets are exhaustively described. The combinations of cisplatin plus paclitaxel with or without bevacizumab is the most active regimen in these clinical setting. Immune-check-point inhibitors and molecularly-targeted agents are promising fields of research.

**Abstract:**

Cervical cancer patients with distant or loco-regional recurrences not amenable by surgery or radiotherapy have limited treatment options, and their 5-year overall survival (OS) rates range from 5% to 16%. The purpose of this paper is to assess the results obtained with chemotherapy and biological agents in this clinical setting. Several phase II trials of different cisplatin (CDDP)-based doublets and a phase III randomized trial showing a trend in response rate, progression-free survival, and OS in favor of CDDP + paclitaxel (PTX) compared with other CDDP-based doublets have been reviewed. The factors predictive of response to chemotherapy as well as the benefits and risks of the addition of bevacizumab to CDDP + PTX have been analyzed. The FDA has recently approved pembrolizumab for patients with recurrent or metastatic cervical cancer in progression on or after chemotherapy whose tumors were PD-L1 positive. Interesting perspectives of clinical research are represented by the use of immune checkpoint inhibitors alone or in addition to chemotherapy, whereas PARP inhibitors and PI3K inhibitors are still at the basic research phase, but promising.

## 1. Introduction 

GLOBOCAN estimates of incidence and mortality worldwide for 36 cancers in 185 countries have detected 569,874 new cases of cervical cancer and 311,365 deaths due to this tumor in 2018 [1]. The primary treatment of early stage disease is either surgery or definitive radiotherapy consisting of pelvic external beam radiotherapy plus brachytherapy [2,3,4]. According to the new 2018 FIGO staging system, tailored abdominal radical hysterectomy with bilateral pelvic lymphadenectomy is the preferred treatment for stage IA1 disease with lymph vascular space involvement (LVSI) and for stage IA2, IB1, IB2 and IIA1 disease, and definitive radiotherapy is an alternative option for patients not fit for surgery or who refuse surgery [2,3,4,5,6]. Concurrent cisplatin (CDDP)-based chemotherapy and radiotherapy (CCRT) plus brachytherapy is the standard of care for patients with locally advanced disease, i.e., in stage IB3-IIA2-IIB-III-IVA [2,3,4,5,6]. Platinum-based neo-adjuvant chemotherapy (NACT) followed by radical hysterectomy has been proposed as an alternative approach [7,8,9,10,11,12]. The achievement of an optimal pathological response is an independent prognostic factor for both disease-free survival (DFS) and overall survival (OS) [9,13]. The combination of paclitaxel (PTX) (175 mg/m^2^ d1) + ifosfamide (IFO) (5 g/m^2^ 24 hour-infusion d1) + CDDP (75 mg/m^2^ d2) (TIP regimen) every 21 days (q21) has obtained the highest optimal pathological response rates, ranging from 43% to 48%, in the neoadjuvant setting [9,11]. Two recent randomized trials showed that CCRT achieves a better DFS and a similar OS compared to NACT followed by radical surgery in patients with 1994 FIGO stage IB2-II cervical cancer [14,15].

Cervical cancer relapses after primary treatment in approximately 10–20% of patients with early stage disease and no evidence of nodal metastases, and up to 64–70% of those with nodal metastases and/or locally advanced disease, and limited treatment options are available for these patients [16,17,18].

Radiotherapy or CCRT is the standard treatment for central or lateral pelvic recurrence in patients primarily treated with radical hysterectomy without adjuvant radiotherapy [16,17,18,19,20]. Radical hysterectomy has been seldom used in patients with small (<2 cm) persistent tumor or centrally located recurrence in the cervix or vaginal fornices after definitive radiotherapy [16,21,22,23,24]. This surgical approach obtained 5-year survival rates ranging from 49% to 84%, witha high rate of severe postoperative complications and especially of fistulas.

Pelvic exenteration with reconstructive procedures usually represents the only therapeutic option with curative intent in accurately selected women with central pelvic recurrence after radiotherapy, with perioperative mortality rates ranging from 1% to 10% and with 5-year survival rates ranging from 21% to 73% [18,25,26,27,28,29,30,31]. Complications have been reported in 49–57% of patients, most commonly fistulas, ureteral strictures, pyelonephritis, wound complications, or bowel obstructions. Some authors have suggested the use of NACT before pelvic exenteration [30].

Lateral pelvic recurrence in patients with prior radiotherapy is usually treated with chemotherapy with palliative intent [18], although Hockel [32] has proposed the use of laterally extended endopelvic resection in highly selected cases.

Radiotherapy or CCRT is recommended in patients with isolated para-aortic recurrence, who can however experience an unfavorable outcome because of systemic spread of disease [33,34,35,36]. Stereotactic body radiation therapy (SBRT), which deliversa much higher radiation dose to the tumor with a steep dose gradient outside the targets, is an interesting therapeutic tool for selected patients with oligometastatic disease, involving not only nodes, but also other sites such as lung, liver, and soft tissues [37,38,39,40,41]. Resection of isolated metastases can be sometimes proposed in selected patients, especially in those with solitary inguinal or lung metastases [42,43].

Pharmacological options for patients with distant or loco-regional recurrences not amenable by surgery or radiotherapy are limited [44,45,46,47]. Five-year OS rates range from 5% to 16% and fewer than 20% of patients survive oneyear.

The purpose of this paper is to assess the results obtained with chemotherapy and biological drugs in this clinical setting and to show the promising perspectives given by novel agentswhich arestill at the basic research phase.

## 2. Single-Agent Chemotherapy

CDDP is the most active agent in this clinical setting, with response rates of 17–38% [5,6,7,8,9,10,11]. Most responses are partial and short-lived, with a median progression free survival (PFS) of approximately 3 months and median OS of 6.5–9 months [48,49,50,51,52,53,54]. Complete responses are predominantly seen in patients with extra-pelvic metastases rather than inthose with pelvic failure.

A Gynecologic Oncology Group (GOG) trial randomly allocated 497 patientsto receive either CDDP 50 mg/m^2^ q21 or CDDP 100 mg/m^2^ q21 or CDDP 20 mg/m^2^ d1–5 q21 [49]. The response rates were 21%, 31%, and 25% respectively, median PFS ranged from 3.7 to 4.6 months, and median OS ranged from 6.1 to 7.1 months. CDDP 100 mg/m^2^ achieved a significantly better response rate than CDDP 50 mg/m^2^, with higher bone marrow and renal toxicity and without any benefit in terms of PFS and OS.

In the experience of Lele and Piver [51], single-agent CDDP obtained an objective response in 4% of central relapses, 33% of liver metastases, 40% of supraclavicular nodal recurrences, and 48% of lung failures.

Several phase II studies have been conducted to identify other active drugs in this clinical setting [55,56,57,58,59,60,61,62,63,64,65,66,67,68,69,70,71,72,73,74,75,76,77,78,79] (Table 1). Higher response rates were obtained for lesions in previously non irradiated areas compared with those in irradiated fields. Median duration of response ranged from 2.5 to 6.5 months, and median OS ranged from 4.2 to 15.2 months. Nab-paclitaxel is a nanoparticle formulation of albumin-bound PTX, which can be delivered without premedication since the risk of hypersensitivity is very low [80]. It is noteworthy that the 29% response rate is the highest ever recorded in the GOG for a single-agent against platinum resistant disease [79]. This drug was equally active in patients who had primarily lymph node metastases or visceral metastases.

## 3. Combination Chemotherapy

In the phase II trials reported in Table 2, the combination of CDDP with other drugs, with additive or synergistic activity and non-overlapping toxicity, obtained response rates of 21–50%, with a median PFS of 4.8–10.5 months and a median OS of 6.4–25+ months [44,81,82,83,84,85,86,87,88,89,90,91,92,93]. These regimens usually achieved higher response rates and longer PFS compared with single-agent CDDP, but at the cost of greater toxicity and with no improvement in OS. Still again, response rates were sharply higher in non-irradiated (57–70%) than in irradiated areas (15–36%).

The GOG179 trial randomized 356 patients with stage IVb or recurrent or persistent squamous andnonsquamous cell cervical carcinoma unsuitable for surgery and/or radiotherapy with curative intent to receive either single-agent CDDP (50 mg/m^2^) q21 or topotecan (0.75 mg/m^2^ d1–3) + CDDP (50 mg/m^2^ d1) q21 or methotrexate + vinblastine + doxorubicin + CDDP (MVAC regimen) every 28 days (q28) [94]. The MVAC arm was early discontinued because of four toxic deaths, principally due to neutropenic sepsis, among 63 patients. CDDP + topotecan achieved a higher response rate (*p* = 0.004), a longer median PFS (adjusted Relative Risk (RR) = 0.738 (95% Confidence Interval (CI) = 0.578–0.942) and a longer OS (adjusted RR = 0.77, 95%CI = 0.600–0.992) compared with single-agent CDDP, associated with anincreased incidence of severe adverse events, and especially of grade 3–4 neutropenia (70% versus 1.4%) and thrombocytopenia (31.3% versus 3.4%) (Table 3). The addition of topotecan to CDDP improved PFS and OS both in patients who had received prior CDDP asradiation sensitizer and in those who had not. GOG179 is the first randomized trial able to demonstrate an OS benefit for CDDP-combination chemotherapy versus single-agent CDDP in this clinical setting.

Monk et al. [95] randomly allocated 513 patients with stage IVB, recurrent, or persistent squamous and nonsquamous cell cervical cancer to undergo either PTX + CDDP or vinorelbine + CDDP or gemcitabine + CDDP or topotecan + CDDP 50 (Table 3). Although the trial was stopped early at a planned interim analysis for futility, there was a trend in response rate, PFS, and OS in favor of CDDP + PTX. The rate of severe neutropenia was approximately 50% with all regimens except gemcitabine + CDDP where it was approximately 15%. There were 11 toxic deaths, but no correlation was found with the chemotherapy regimen.

A randomized phase III trial, including 253 patients with recurrent or metastatic squamous and nonsquamous cell cervical cancer who had undergone one or less one platinum-based-treatment and no prior taxane, demonstrated a noninferiority in terms of OS of the combination of PTX + carboplatin (CBDCA) compared with PTX + CDDP [96] (Table 3). However, among the patients who had not received prior CDDP, median OS was worse for PTX + CBDCA arm (13.0 versus 23.2 months; Hazard Ratio (HR) = 1.571; 95%CI = 1.062–2.324).

CDDP-based three- or four-drug regimens obtained no clear improvement in the clinical outcome compared with CDDP-based doublets [98,99,100,101,102]. In a randomized trial enrolling 287 patients with advanced, recurrent, or persistent squamous cell cervical carcinoma, the addition of bleomycin (30 U over 24 h) to CDDP (50 mg/m^2^) + IFO (5 g/m^2^ over 24 h) q21 did not change response rate, PFS, and OS [98]. Whereas TIP appears to be the most active regimen as NACT followed by radical surgery, this combination chemotherapy achieved response rates of 46–67% and a median OS of 6–19 months in patients with recurrent, persistent, or metastatic disease, similar to those obtained with CDDP-based doublets [99,100,101]. Response rates ranged from 36% to 52% in irradiates sites and from 60% to 75% in areas outside the previous radiotherapy fields. The excision repair cross-complement 1 (ERCC1) status appears to be a predictive and prognostic factor in this clinical setting. This nucleotide excision repair gene is involved in resistance to platinum compounds in different malignancies [103,104,105,106,107]. A study performed on 45 tissue samples from patients with recurrent or metastatic cervical cancer treated with CDDP + IFO with or without PTX, reported that high ERCC1 expression was an independent poor predictor of both PFS (HR = 2.473, 95% CI = 1.146–5.339) and OS (HR = 3.187, 95% CI = 1.346–7.546) [106]. In another study on 32 women with recurrent or metastatic disease undergoing platinum-based chemotherapy, the patients with high ERCC1 expression experienced a lower response rate (15% versus 74%, *p* = 0.001), a shorter PFS (HR = 2.428; 95%CI =1.145–5.148), and a shorter OS (HR = 2.322; 95% CI = 1.051–5.29) compared to those with low ERCC1 expression [106].

The analysis of 428 advanced cervical cancer patients treated with a CDDP-based regimen in the GOG trials 110 [87], 169 [44] and 179 [94] detected that five factors, i.e., Black women (Odds Ratio(OR) = 0.49, 95%CI = 0.28–0.83), performance status>0 (OR= 0.60, 95%CI = 0.38–0.94), pelvic disease (OR = 0.58, 95%CI = 0.38–0.90), prior radiosensitizing chemotherapy (OR= 0.52, 95%CI = 0.32–0.85), and time interval from diagnosis to first recurrence <1 year (OR = 0.61, 95%CI = 0.39–0.95) were independent predictors of poor response to treatment [108]. The patients were classified to be at low-risk, mild-risk, or high-risk, according to whether they had ≤1 factor, 2–3 factors, or 4–5 factors, and the corresponding median PFS and OS were 6.34 months and 11.10 months, 4.60 months and 9.17 months, and 2.79 months and 5.49 months, respectively. This predictive model was externally validated using GOG trial 149 [98] data that had comparable patient characteristics.

## 4. Bevacizumab

In a phase II GOG trial, single-agent bevacizumab (BEV) (15 mg/kg q21) was administered to 46 patients with persistent or recurrent squamous cell carcinoma of the cervix [109]. Eleven patients (24%) survived progression free for at least 6 months, and 5 patients (11%); experienced partial responses. The median response duration was 6.21 months, and median PFS and OS for all patients were 3.40 months and 7.29 months, respectively.

Four-hundred and fifty-two patients with recurrent, persistent, or metastatic squamous and nonsquamous cell cervical cancer enrolled in the GOG 240 bi-factorial trial were randomized to receive chemotherapy (CDDP+ PTXor topotecan + PTX) with or without BEV (Table 3) [97]. The combination of topotecan + PTX was chosen on the basis of both laboratory data, detecting a synergistic anticancer activity when the two drugs were administered sequentially [110], and clinical data from a phase II study showing that this regimen was generally well tolerated and active [111]. In the GOG 240 trial, topotecan + PTX (either with or without BEV) had a higher risk of progression (HR = 1.39; 95%CI = 1.09–1.77) and a trend to higher risk of death (HR = 1.20; 99%CI = 0.82–1.76) compared to CDDP + PTX (either with or without BEV) [97]. The addition of BEV to chemotherapy (with two regimens combined) significantly improved response rates (*p* = 0.008), PFS (HR = 0.67, 95%CI = 0.54–0.82), and OS (HR = 0.71; 98%CI = 0.54–0.95). CDDP +PTX + BEV had a HR for death of 0.68 (95%CI = 0.48–0.97) compared with CDDP + PTX, and topotecan + PTX + BEV had a HR for death of 0.74(95%CI = 0.53–1.05) compared with topotecan + PTX. The incorporation of BEV significantly increased the rates of grade≥2hypertension (25% versus 2%), grade ≥3 gastrointestinal or genitourinary fistulas (6% versus 0%), and grade ≥3 thrombo-embolic events (8% versus 1%).

An update of the study with a longer follow-up confirmed that chemotherapy + BEV was associated with a longer OS compared with chemotherapy alone (HR = 0.77,95%CI = 0.62–0.95) [47]. There was no negative rebound after progression while receiving BEV, since post-progression survival was not significantly different between the patients treated with chemotherapy + BEV and those treated with chemotherapy alone. These results represent the proof-of-concept of the efficacy of anti-angiogenesis therapy in cervical cancer.

Other studies have confirmed the efficacy of BEV combined with CDDP + PTX in patients with persistent, recurrent, or metastatic carcinoma of the uterine cervix [112,113]. According to a systematic review of 23 studies, CDDP + PTX + BEV and topotecan + PTX + BEV were likely to prolong OS compared with non–BEV-containing therapies, and CDDP + PTX + BEV had the highest probability of being the most efficacious regimen [112]. Response rates and fistula rates with CBDCA + PTX + BEV are similar to those reported with CDDP + PTX + BEV [114,115]. The risks and benefits of the addition of BEV to chemotherapy should be exhaustively discussed with the patient herself, taking into account the increased probability of fistula formation, especially in the case of locally persistent or recurrent disease after radiotherapy or CCRT [116,117,118]. Hypoalbuminemia is an additional risk factor for fistula formation [119].

Tewari et al. [120] retrospectively classified the patients enrolled in the GOG 240 trial in three subgroups based on the predictive factors of poor response to chemotherapy suggested by Moore et al. [108]. The application of the Moore’s criteria classified most patients in the mid-risk subset (67%), whereas low-risk and high-risk patients accounted for 19% and 14%, respectively, of the entire population. The HRs of death for treating with BEV in low-risk, mid-risk, and high-risk subgroups were 0.96 (95% CI = 0.51–1.83; *p* = 0.9087), 0.673 (95%CI = 0.5–0.91; *p* = 0.0094), and 0.536 (95%CI = 0.32–0.905; *p* = 0.0196), respectively. Therefore, BEV could be avoided in previously irradiated low-risk patients, since the OS advantage given by the addition of this antiangiogenic agent is not significant.

## 5. Immune Checkpoint Inhibitors 

Recent data seem to suggest a promising role for immune checkpoint inhibitors in the treatment of patients with advanced carcinoma of the uterine cervix [121,122,123,124].

The anti-programmed death [PD]-1 antibody nivolumab (240 mg every 14 days) obtained an objective response in 26% of 19 patients with recurrent or metastatic cervical cancer and in 20% of 5 patients with recurrent or metastatic vaginal/vulvar cancer [122]. As for the former, the median duration of response was not yet reached after a median follow-up of 19.2 months.

The phase II KEYNOTE-158 study, investigating the anti- PD-1 antibody pembrolizumab (200 mg q21) in several cancer types, included 98 patients with pretreated advanced squamous and nonsquamous cell cervical cancer [123]. Of these 82 had PD-ligand 1 (PD-L1)-positive tumors, according to the PD-L1 IHC 22C3 pharmDx assay, and 77 had undergone more than one chemotherapy line for recurrent or metastatic disease. The measure of PD-L1 expression was the combined positive score (CPS), defined as the ratio of PD-L1–positive cells (tumor cells, lymphocytes, and macrophages) to the total number of tumor cells × 100. A tumor was considered to be PD-L1 positive if CPS was >1. There were 3 complete responses and 9 partial responses, with an overall response rate of 12%, and all 12 responses were detected in patients with PD-L1–positive tumors. Nine of the 12 responses were still ongoing after >9 months of follow-up. The safety profile was the same previously reported for pembrolizumab in patients with advanced cancer, and no novel adverse event occurred. Based on these results, the Food and Drug Administration (FDA) has approved pembrolizumab for patients with recurrent or metastatic cervical cancer in progression on or after chemotherapy whose tumors were PD-L1 positive.

Several trials of immune checkpoint inhibitors are currently ongoing in metastatic, recurrent, or persistent cervical cancer (Table 4). One of the most interesting is the phase III, randomized BEATcc (NCT03556839) trial which will enroll a total of 404 women with squamous and nonsquamous cell carcinoma of the uterine cervix [125]. Patients will be randomly allocated to receive either CCDP (50 mg/m^2^) + PTX (175 mg/m^2^) + BEV (15 mg/kg) q21 or the same regimen plus atezolizumab (1200 mg q21). Complete responders after ≥6 cycles will be allowed to continue only on biologic therapy, i.e., BEV or BEV + atezolizumab. OS is the primary endpoint of the study which is estimated to close in 2022.

## 6. Other Agents 

### 6.1. Phosphatidylinositol 3-Kinase (PI3K)/AKT/Mammalian Target of Rapamycin (mTOR)Inhibitors

#### 6.1.1. Basic Research 

Activation of phosphatidylinositol 3-kinase (PI3K)/AKT/mammalian target of rapamycin (mTOR) pathway sometimes occurs in cervical cancer [126,127,128,129]. PIK3catalytic subunit α (PI3KCA) mutations have been found in 14–25% of adenocarcinomas and in 37.5–48% of squamous cell carcinomas of the uterine cervix [126,128].

PI3K pathway was strongly activated in PTX-resistant HeLa and ME180 cell lines established from metastatic sites of cervical cancer compared to parental cells, and the combination of PTX and a PI3K inhibitor revealed a synergistic antiproliferative activity by enhancing PTX–induced S and G2/M arrest in PTX-resistant cell lines [130]. Therefore, therapeutic strategies targeting PI3K pathway could revert the chemoresistance of cervical cancer cells to PTX and could provide an promising research perspective for the management of patients with this malignancy.

#### 6.1.2. In Human Studies 

A retrospective study conducted on 82 squamous and nonsquamous cell cervical cancer patients treated with CCRT showed that PIK3CA mutational status correlated with a worse OS (HR = 6.0; 95%CI = 2.1–17.5) in FIGO stage Ib /II disease, but not in stage II–IVa disease (HR = 1.0, 95%CI = 0.32–3.1) [127]. Very few and conflicting clinical data are currently available in the literature as for the use of PI3K/AKT/mTOR pathway targeted agents [128,131,132]. A phase II trial of temsirolimus (25 mg iv q28) reported a partial response in one (3%) and a stable disease in 19 (58%) of 33 patients with recurrent, unresectable locally advanced, or metastatic squamous and nonsquamous cell carcinoma of the uterine cervix [131]. The single patient with a partial response experienced no evidence of progression for 13.9 months, whereas the median duration of stable disease was 6.5 months with no correlation with PTEN and PIK3CA status. A phase I clinical study on 55 patients with metastatic or recurrent squamous and nonsquamous cell cervical cancer, of which 22 had PIK3CA mutations and/or PTEN loss, found that those patients treated with PI3K/AKT/mTOR targeted agents (such as temsirolimus) matching the aberrations in this pathway achieved a longer median PFS (6.0 months versus 1.5 months, *p* = 0.026) than those who did not receive such matched therapy [128]. Conversely, in a phase II trial, the combination of the pan-AKT inhibitor GSK2141795 and the MEK inhibitor trametinibobtained no confirmed response in 14 patients with squamous and nonsquamous cell persistent or recurrent carcinoma of the uterine cervix [133].

### 6.2. Poly (Adenosine diphosphate (ADP)-Ribose) Polymerase (PARP)Inhibitors (PARPi)

#### 6.2.1. Basic Research

Poly (adenosine diphosphate (ADP)-ribose) polymerase (PARP) inhibitors (PARPi) exert anti-proliferative effects on human cervical cancer cell lines [132,134,135]. PARPi are still at the basic research phase, but promising. Bianchi et al. [134] reported that none of 9 primary cervical cancer cell lines had homologous recombination (HR) deficiency, but 3 of these showed strong PARP protein activity, i.e., PAR expression, and were very high sensitive to olaparib in vitro. This PARPi caused cell cycle arrest in the G2/M phase and induced apoptosis. In vivo antitumor activity of olaparib was tested in severe combined immunodeficiency (SCID) mice who were given a subcutaneous injection of human cervical cancer cells. Animals treated with olaparib experienced a slower tumor growth and a prolonged survival compared to controls. PAR expression might be a novel biomarker able to identify a subset of cervical cancer patients who could benefit from PARPi.Additional studies with PARPi alone or combined with other agents are strongly warranted, especially in patients with radiotherapy and/or chemotherapy-resistant disease.

The α-specific PI3K inhibitor alpesilib and the PARPi talazoparib synergized to inhibit cervical cancer cell proliferation, migration, and invasion in vitro and in vivo [135]. Cancer cells with aberrant PI3K activation were more responsive to these combined agents. Besides catalytic activity, talazoparib trapped PARP on damaged DNA and induced cytotoxic effects. Alpesilib could co-operate with talazoparibto increase PARP trapping on chromatin andto induce severe DNA damage.

#### 6.2.2. In Human Studies

Roszik et al. [136] found a significantly higher expression of DNA repair genes, especially those involved in HRand mismatch repair pathways, in tumor tissues compared with adjacent normal tissues from 28 patients with recurrent cervical cancer who underwent pelvic exenteration. High-risk HPV E6 and E7 reduced the ability of the HR pathway to complete double-strand break repair by approximately 50%, thus leading to HR deficiency [137]. The combination of immune checkpoint inhibitors and PARPi should be investigated in patients with recurrent cervical cancer. NCT04068753 is an ongoing phase II trial of niraparib in combination with the anti-PD-1 antibody dostarlimab in this clinical setting.

## 7. Conclusions

Cervical cancer patients with distant or loco-regional recurrences not amenable by surgery or radiotherapy have a very poor prognosis, and pharmacological treatment options have only a palliative intent.CDDP-baseddoublets have obtained response rates of 21–50%, with a median PFS of 4.8–10.5 months and a median OS of 6.4–25+ months in this clinical setting. A phase III randomized trial has shown a trend in response rate, PFS and OS in favor of CDDP + PTX compared with CDDP+ vinorelbine, CDDP+ gemcitabine or CDDP + topotecan. The addition of BEV to CDDP + PTX significantly improved PFS and OS, but increased the risk of adverse events and especially of grade >3 gastrointestinal or genitourinary fistulas. BEV could be avoided in previously irradiated low-risk women according to Moore’s criteria, where the OS benefit is small. Pembrolizumab has been approved by the FDA for patients with recurrent or metastatic cervical cancer in progression on or after chemotherapy whose tumors were PD-L1 positive, and several interestingtrials with immune checkpoint inhibitors are currently ongoing. PARP inhibitors and PI3K inhibitors are still at the basic research phase, but promising.

## Figures and Tables

**Table 1:** Single agentactivity in metastatic, persistent, or recurrent carcinoma of the uterine cervix.

Agent	Author	Dose	Histology	Patients	Response Rate
CBDCA	Areseneau [55] **	340–400 mg/m^2^ q28	S	39	28%
	McGuire [56] **	340–400 mg/m^2^ q28	S	175	15%
	Weiss [57] **	400 mg/m^2^ q28	S	41	15%
IFO	Meanwell [58]	5 g/m^2^ q21	S	30	33%
	Sutton [59] *	1.2 g/m^2^ d1–5 q28	S	30	11%
	Sutton [60] *	1.2–1.5 g/m^2^ d1–5 q28	NS	40	15%
VNR	Morris [61] **	30 mg/m^2^ q7	S	33	18%
	Lhommè [62] **	30 mg/m^2^ q7	S, NS	41	17%
	Muggia [63] *	30 mg/m^2^ d1, 8 q21	NS	28	7%
GEM	Schilder [64] *	800 mg/m^2^ d1, 8, 15 q28	S	27	8%
	Schilder [65] *	800 mg/m^2^ d1, 8, 15 q28	NS	19	4.5%
PTX	McGuire [66] **	135–170 mg/m^2^ q21	S	52	17%
	Kudelka [67] **	250 mg/m^2^ q21 (+GCSF)	S	32	25%
	Curtin [68] *	135–170 mg/m^2^ q21	NS	42	31%
DCX	Garcia [69] *	100 mg/m^2^ q21	S	23	9%
	Pearl [70] *	35 mg/m^2^ d1, 8, 15 q28	S, NS	10	0%
Topotecan	Bookman [71] *	1.5 mg/m^2^ d1–5 q21	S	40	12.5%
	Muderspach [72] **	1.5 mg/m^2^ d1–5 q28	S	43	19%
Irinotecan	Verschraegen [73] *	125 mg/m^2^ × 4 wks q42	S	42	21%
	Lhommè [74] **	350 mg/m^2^ q21	S	51	16%
Pemetrexed	Goedhals [75] **	500–600 mg/m^2^ q21	S	34	18%
	Miller [76] *	900 mg/m^2^ q21	S, NS	27	15%
Altretamine	Rose [77] *	260 mg/m^2^ q21	S	26	0%
Oral VP-16	Rose [78] *	40–50 mg/m^2^/d × 21d q28	S	17	12%
Nab-PTX	Alberts [79] *	125 mg/m^2^ d1, 8, 15 q28	S, NS	35	29%

* prior chemotherapy; ** no prior chemotherapy (except as a radiation sensitizer). Legend: CBDCA, carboplatin; S, squamous cell carcinoma; IFO, ifosfamide; NS, non squamous cell carcinoma; VNR, vinorelbine; GEM, gemcitabine; PTX, paclitaxel; DCX, docetaxel; VP-16, etoposide; Nab-PTX, Nab-paclitaxel.

**Table 2:** Phase II trials of cisplatin-based doublets in metastatic, persistent, or recurrent carcinoma of the uterine cervix.

Agent	Author	Dose	Histology	Patients	Response Rate
CDDP + 5-FU	Bonomi [81] *	CDDP 50 mg/m^2^ d1 + 5FU 1000 mg/m^2^ q21	S	5	22%
	Kaern [82] *	CDDP 100 mg/m^2^ d1 + 5FU 1000 mg/m^2^ d 1–5 q21	S	32	47%
CDDP + CAPE	Benjapibal [83] *	CDDP 50 mg/m^2^ d1 + CAPE 1000 mg/m^2^ bid d1–4 q21	S, NS	16	50%
CDDP + BLEO	Daghestani [84] ^	CDDP 120 mg/m^2^ d1 + BLEO 10 mg/m^2^ bolus d1 + BLEO 10 mg/m^2^ d1–5 or 1–7 q21–28	S, NS	24	54%
CDDP + IFO	Coleman [85] *	CDDP 50 mg/m^2^ d1 + IFO 1.5 g/m^2^ d 1–5 q21	S	42	38%
	Cervellino [86] *	CDDP 20 mg/m^2^ d1-5 + IFO 2.5 g/m^2^ d 1–5 q28	S	30	50%
	Omura [87] *	CDDP 50 mg/m^2^ d1 + IFO 5 g/m^2^ 24 h q21	S	151	31%
CDDP + GEM	Burnett [88] *	CDDP 50 mg/m^2^ d1 + GEM 1250 mg/m^2^ d1, 8 q21	S	17	42%
CDDP + PTX	Rose [89] *	CDDP 75 mg/m^2^ d2 + PTX 135 mg/m^2^ 24 h d 1 q21	S	41	46%
	Papadimitriou [90]*	CDDP 75 mg/m^2^ d1 + PTX 175 mg/m^2^ d1 q21 + G-CSF	S, NS	34	47%
	Piver [91] *	CDDP 75 mg/m^2^ d2 + PTX 135 mg/m^2^ 24 h d1 q28	S, NS	20	45%
	Moore [44] *	CDDP 50 mg/m^2^ d2 + PTX 135 mg/m^2^ 24 h d1 q21	S	130	38%
CDDP + VNR	Gebbia [92] *	CDDP 80 mg/m^2^ d1 + VNR 25 mg/m^2^ d1, 8 q21	S, NS	42	48%
CDDP + tirapazamine	Smith [93] *	CDDP 75 mg/m^2^ d1 + tirapazamine 260 mg/m^2^ q21	S, NS	53	32%

* no prior chemotherapy; ^ prior chemotherapy in 2 patients.Legend: CDDP, cisplatin; 5-FU, 5-fluorouracil; S, squamous cell carcinoma; CAPE, capecitabine; NS, non-squamous cell carcinoma; BLEO, bleomycin; IFO, ifosfamide; GEM, gemcitabine; PTX, paclitaxel; G-CSF, granulocyte-colony-stimulating factor; VNR, vinorelbine.

**Table 3:** Phase III trials of cisplatin-based chemotherapy in metastatic, persistent, or recurrent carcinoma of the uterine cervix.

Author	Agent	Patients	RR	Median PFS	Median OS
Long [94]	CDDP 50 mg/m^2^ d1 q21	146	13%	2.9 months	6.5 months
	CDDP 50 mg/m^2^ d1 +TOP 0.75 mg/m^2^ d 1–3 q21	147	27% *p* = 0.004	4.6 months *p* = 0.0075	9.4 months *p* = 0.021
Monk [95]	PTX 135 mg/m^2^ (24 h) d1+ CDDP 50 mg/m^2^ d2 q21	103	29.1%	5.82 months	12.87 months
	VNR 30 mg/m^2^ d1, 8 + CDDP50 mg/m^2^ d1 q21	108	25.9% *	3.98 months **	9.99 months ***
	GEM 1000 mg/m^2^ d1,8 + CDDP 50 mg/m^2^ d1 q21	112	22.3% *	4.70 months **	10.28% months ***
	TOP 0.75 mg/m^2^ d1–3+ CDDP 50 mg/m^2^ d1 q21	111	23.4% *	4.57 months **	10.25 months ***
Kitagawa [96]	PTX 135 mg/m^2^ (24 h) d1 + CDDP 50 mg/m^2^ d2 q21	127	58.8%	6.9 months	18.3 months
	PTX 175 mg/m^2^ (3 h) d1 + CBDCA AUC5 d1 q21	126	62.6% *p* = 0.665	6.2 months ^	17.5 months ^^
Tewari [97]	CDDP 50 mg/m^2^ d1+ PTX 135–175 mg/m^2^ d1 ± BEV 15 mg/kg d1 q21 TOP 0.75 mg/m^2^ d1–3 + PTX 175 mg/m^2^ d1 ± BEV 15 mg/kg q 21		48%	8.2 months	17 months
	BEV + CT (the two regimen combined) CT alone (the two regimen combined)		36% *p*= 0.008	5.9 months *p*= 0.002	13.3 months *p* = 0.004

* OR (95%CI) for reference arm (PTX + CDDP) to other doublets: VNR + CDDP, 1.17 (0.54–2.58); GEM + CDDP, 1.43 (0.65–3.19); TOP + CDDP, 1.34 (0.61–2.98); ** HR (95%CI) for reference arm (PTX +CDDP) to other doublets: VNR + CDDP, 1.36 (0.97–1.90); GEM + CDDP, 1.39 (0.99–1.96); TOP + CDDP, 1.27 (0.90–1.78); *** HR (95%CI) for reference arm (PTX + CDDP) to other doublets: VNR + CDDP, 1.15 (0.79–1.67); GEM + CDDP, 1.323 (0.91–1.92); TOP + CDDP, 1.26 (0.86–1.82); ^ HR: 1.041 (95%CI = 0.803–1.351); ^^ HR: 0.994 (90%CI = 0.789–1.253); Legend: RR, response rate; PFS, progression free survival; OS, overall survival; CDDP, cisplatin; TOP, topotecan; PTX, paclitaxel; VNR, vinorelbine; GEM, gemcitabine; CBDCA, carboplatin; AUC, area under curve; BEV, bevacizumab; CT, chemotherapy; OR, Odds Ratio; CI, confidence interval; HR, Hazard Ratio.

**Table 4:** Ongoing trials with immune checkpoint inhibitors in metastatic, recurrent, or persistent carcinoma of the uterine cervix.

NCT Number	Trial
NCT02257528	A phase II evaluation of Nivolumab, a fully human antibody against PD-1, in the treatment of persistent or recurrent cervical cancer
NCT03972722	An open, multi-center, single-arm phase II clinical study to evaluate the efficacy and safety of recombinant fully human anti-PD-1 monoclonal antibody (GLS-010 injection) in patients with recurrent or metastatic cervical cancer
NCT03104699	A phase 1/2, open-label, multiple ascending dose trial to investigate the safety, tolerability, pharmacokinetics, biological, and clinical activity of AGEN2034 Balstilimab, anti-PD-1 antibody) in subjects with metastatic or locally advanced solid tumors, with expansion to second line cervical cancer
NCT03808857	Phase II clinical study to evaluate the efficacy and safety of GB226 (Genolimzumab, anti-PD-1 antibody) in treatment of recurrent or metastatic cervical cancer patients with PD-L1 positive who failed in platinum-based chemotherapy
NCT03676959	A clinical study of PD-L1 antibody ZKAB001 (Drug Code) in recurrent or metastatic cervical cancer. An open-label, dose-escalation, bi-weekly phase I clinical trial in treating patients with recurrent or metastatic cervical cancer
NCT01693783	A phase 2 study of Ipilimumab (anti-CTLA antibody) in women with metastatic or recurrent HPV-related cervical carcinoma of either squamous cell or adenocarcinoma histologies
NCT03894215	A two-arm, randomized, non-comparative, phase 2 trial of AGEN2034 (Balstilimab, anti-PD-1 antibody) as a monotherapy or combination therapy with AGEN1884 (Zalifrelimab, anti-CTLA antibody) or with placebo in women with recurrent cervical cancer (Second Line) RaPiDS
NCT03495882	A phase 1/2, open-label, multi-arm trial to investigate the safety, tolerability, pharmacokinetics, biological, and clinical activity of AGEN1884 (Zalifrelimab, anti-CTLA4 antibody) in combination with AGEN2034 (Balstilimab, Anti-PD-1 antibody) in subjects with metastatic or locally advanced solid tumors and expansion into select solid tumors (cervical)
NCT03816553	SHR-1210 (Camrelizumab), a novel anti-PD-1 antibody, in combination with apatinib ^1^ in patients with metastatic, persistent or recurrent cervical cancer: a single-arm, open label, multi-center, phase II study
NCT03826589	Avelumab (anti-PD-L1 antibody) with Axitinib ^2^ in persistent or recurrent cervical cancer after platinum-based chemotherapy a proof-of-concept study (ALARICE Study)
NCT03912415	An international randomized double-blind clinical trial of BCD-100 (Prolgolimab anti-PD-1 antibody) plus platinum-based chemotherapy with and without Bevacizumab versus placebo plus platinum-based chemotherapy with and without Bevacizumab as first-line treatment of subjects with advanced cervical cancer
NCT03635567	A phase 3 randomized, double-blind, placebo-controlled trial of Pembrolizumab (anti- PD1- antibody) (MK-3475) plus chemotherapy versus chemotherapy plus placebo for the first-line treatment of persistent, recurrent, or metastatic cervical cancer (KEYNOTE-826)
NCT03257267	An open-label, randomized, phase 3 clinical trial of REGN2810 (Cemiplimab, anti- PD-1antibody) versus investigator’s choice of chemotherapy (pemetrexed, gemcitabine, topotecan, irinotecan, or vinorelbine) in recurrent or metastatic cervical carcinoma
NCT03228667	QUILT-3.055: a phase IIb, single-arm, multicohort, open-label study of ALT-803 ^3^ in combination with PD-1/PD-L1 checkpoint inhibitor in patients who have disease progression following an initial response to treatment with PD-1/PD-L1 checkpoint inhibitor therapy (several types of tumor including cervical cancer)
NCT02921269	A phase 2 study of Atezolizumab (MPDL3280A, anti- PD-L1 antibody) in combination with Bevacizumab in patients with recurrent, persistent or metastatic cervical cancer
NCT03556839	A randomized phase III trial of platinum chemotherapy plus paclitaxel with Bevacizumab and Atezolizumab (anti-PD-L1 antibody) versus platinum chemotherapy plus paclitaxel and Bevacizumab in metastatic (stage IVB), persistent or recurrent carcinoma of the cervix (BEATcc)

Legend ^1^ tyrosine kinase inhibitor that selectively inhibits the vascular endothelial growth factor receptor (VEGFR)-2, ^2^ tyrosine kinase inhibitor that selectively inhibits VEGFR-1, -2 and -3. ^3^ interleukin (IL)-15 superagonist.

## References

[1] Bray F., Ferlay J., Soerjomataram I., Siegel R.L., Torre L.A., Jemal A. (2018). Global cancer statistics 2018: GLOBOCAN estimates of incidence and mortality worldwide for 36 cancers in 185 countries. CA Cancer J. Clin..

[2] Marth C., Landoni F., Mahner S., McCormack M., Gonzalez-Martin A., Colombo N. (2018). ESMO guidelines committee cervical cancer: ESMO clinical practice guidelines for diagnosis, treatment and follow-up. Ann. Oncol..

[3] Cibula D., Pötter R., Planchamp F., Avall-Lundqvist E., Fischerova D., Haie Meder C., Köhler C., Landoni F., Lax S., Lindegaard J.C. (2018). The European Society of Gynaecological Oncology/European Society for Radiotherapy and Oncology/European Society of Pathology guidelines for the management of patients with cervical cancer. Radiother. Oncol..

[4] Koh W.J., Abu-Rustum N.R., Bean S., Bradley K., Campos S.M., Cho K.R., Chon H.S., Chu C., Clark R., Cohn D. (2019). Cervical Cancer, Version 3.2019, NCCN clinical practice guidelines in oncology. J. Natl. Compr. Cancer Netw..

[5] Landoni F., Maneo A., Colombo A., Placa F., Milani R., Perego P., Favini G., Ferri L., Mangioni C. (1997). Randomised study of radical surgery versus radiotherapy for stage Ib-IIa cervicalcancer. Lancet.

[6] Ramirez P.T., Frumovitz M., Pareja R., Lopez A., Vieira M., Ribeiro R., Buda A., Yan X., Shuzhong Y., Chetty N. (2018). Minimally Invasive versus Abdominal Radical Hysterectomy for Cervical Cancer. N. Engl. J. Med..

[7] Benedetti-Panici P., Greggi S., Colombo A., Amoroso M., Smaniotto D., Giannarelli D., Amunni G., Raspagliesi F., Zola P., Mangioni C. (2002). Neoadjuvant chemotherapy and radical surgery versus exclusive radiotherapy in locally advanced squamous cell cervical cancer: Results from the Italian multicenter randomized study. J. Clin. Oncol..

[8] Selvaggi L., Loizzi V., DIGilio A.R., Nardelli C., Cantatore C., Cormio G. (2006). Neoadjuvant chemotherapy in cervical cancer: A 67 patients experience. Int. J. Gynecol. Cancer.

[9] Buda A., Fossati R., Colombo N., Fei F., Floriani I., Gueli Alletti D., Katsaros D., Landoni F., Lissoni A., Malzoni C. (2005). Randomized trial of neoadjuvant chemotherapy comparing paclitaxel, ifosfamide, and cisplatin with ifosfamide and cisplatin followed by radical surgery in patients with locally advanced squamous cell cervical carcinoma: The SNAP01 (Studio Neo-Adjuvante Portio) Italian Collaborative Study. J. Clin. Oncol..

[10] Chen H., Liang C., Zhang L., Huang S., Wu X. (2008). Clinical efficacy of modified preoperative neoadjuvant chemotherapy in the treatment of locally advanced (stage IB2 to IIB) cervical cancer: Randomized study. Gynecol. Oncol..

[11] Lissoni A.A., Colombo N., Pellegrino A., Parma G., Zola P., Katsaros D., Chiari S., Buda A., Landoni F., Peiretti M. (2009). A phase II, randomized trial of neo-adjuvant chemotherapy comparing a three-drug combination of paclitaxel, ifosfamide, and cisplatin (TIP) versus paclitaxel and cisplatin (TP) followed by radical surgery in patients with locally advanced squamous cell cervical carcinoma: The Snap-02 Italian Collaborative Study. Ann. Oncol..

[12] Loizzi V., Del Vecchio V., Crupano F.M., Minicucci V., Fumarulo V.V., Resta L., Vimercati A., Bettocchi S., Cicinelli E., Cormio G. (2018). A phase II study: Dose-densec arboplatin and paclitaxel as neoadjuvant chemotherapy in locally advanced cervical cancer. J. Chemother..

[13] Gadducci A., Sartori E., Maggino T., Zola P., Cosio S., Zizioli V., Lapresa M., Piovano E., Landoni F. (2013). Pathological response on surgical samples is an independent prognostic variable for patients with Stage Ib2-IIb cervical cancer treated with neoadjuvant chemotherapy and radical hysterectomy: An Italian multicenter retrospective study (CTF Study). Gynecol. Oncol..

[14] Gupta S., Maheshwari A., Parab P., Mahantshetty U., Hawaldar R., Sastri Chopra S., Kerkar R., Engineer R., Tongaonkar H., Ghosh J. (2018). Neoadjuvantchemotherapy followed by radical surgery versus concomitant chemotherapy and radiotherapy in patients with stage Ib2, IIa, or IIb squamous cervical cancer: A randomized controlled trial. J. Clin. Oncol..

[15] Kenter G., Greggi S., Vergote I., Katsaros D., Kobierski J., Massuger L., van Doorn H.C., Landoni F., Van Der Velden J., Reed N.S. (2019). Results from neoadjuvant chemotherapy followed by surgery compared to chemoradiation for stage Ib2-IIb cervical cancer, EORTC 55994. J. Clin. Oncol..

[16] Leitao M.M., Chi D.S. (2002). Recurrent cervical cancer. Curr. Treat Options Oncol..

[17] Friedlander M., Grogan M.U.S. (2002). Preventative Services Task Force. Guidelines for the treatment of recurrent and metastatic cervical cancer. Oncologist.

[18] Gadducci A., Tana R., Cosio S., Cionini L., Gadducci A., Tana R., Cosio S., Cionini L. (2010). Treatment options in recurrent cervical cancer (Review). Oncol. Lett..

[19] Yin Y.J., Li H.Q., Sheng X.G., Du X.L., Wang C., Lu C.H., Pan C.X. (2015). The treatment of pelvic locoregional recurrence of cervical cancer after radical surgery with intensity-modulated radiation therapy compared with conventional radiotherapy: A retrospective study. Int. J. Gynecol. Cancer.

[20] Van der Velden J., Mom C.H., van Lonkhuijzen L., Tjiong M.Y., Westerveld H., Fons G. (2019). Analysis of isolated loco-regional recurrence rate in intermediate risk early cervical cancer after a type C2 radical hysterectomy without adjuvant radiotherapy. Int. J. Gynecol. Cancer.

[21] Rutledge S., Carey M.S., Prichard H., Allen H.H., Kocha W., Kirk M.E. (1994). Conservative surgery for recurrent or persistent carcinoma of the cervix following irradiation: Is exenteration always necessary?. Gynecol. Oncol..

[22] Coleman R.L., Keeney E.D., Freedman R.S., Burke T.W., Eifel P.J., Rutledge F.N. (1994). Radical hysterectomy for recurrent carcinoma of the uterine cervix after radiotherapy. Gynecol. Oncol..

[23] Maneo A., Landoni F., Cormio G., Colombo A., Mangioni C. (1999). Radical hysterectomy for recurrent or persistent cervical cancer following radiation therapy. Int. J. Gynecol. Cancer.

[24] Mabuchi Kozasa K., Kimura T. (2017). Radical hysterectomy after radiotherapy for recurrent or persistent cervical cancer. Int. J. Gynaecol. Obstet..

[25] Morley G.W., Hopkins M.P., Lindenauer S.M., Roberts J.A. (1989). Pelvic exenteration, University of Michigan: 100 patients at 5 years. Obstet. Gynecol..

[26] Marnitz S., Köhler C., Müller M., Behrens K., Hasenbein K., Schneider A. (2006). Indications for primary and secondary exenterations in patients with cervical cancer. Gynecol. Oncol..

[27] Goldberg G.L., Sukumvanich P., Einstein M.H., Smith H.O., Anderson P.S., Fields A.L. (2006). Total pelvic exenteration: The Albert Einstein College of Medicine/Montefiore Medical Center Experience (1987 to 2003). Gynecol. Oncol..

[28] Berek J.S., Howe C., Lagasse L.D., Hacker N.F. (2005). Pelvic exenteration for recurrent gynecologic malignancy: Survival and morbidity analysis of the 45-year experience at UCLA. Gynecol. Oncol..

[29] Fleisch M.C., Pantke P., Beckmann M.W., Schnuerch H.G., Ackermann R., Grimm M.O., Bender H.G., Dall P. (2007). Predictors for long-term survival after interdisciplinary salvage surgery for advanced or recurrent gynecologic cancers. J. Surg. Oncol..

[30] Landoni F., Zanagnolo V., Rosenberg P.G., Lopes A., Radice D., Bocciolone L., Aletti G., Parma G., Colombo N., Maggioni A. (2013). Neoadjuvant chemotherapy prior to pelvic exenteration in patients with recurrent cervical cancer: Single institution experience. Gynecol. Oncol..

[31] Sardain H., Lavoué V., Foucher F., Levêque J. (2016). Curative pelvicexenteration for recurrentcervical carcinoma in the era of concurrent chemotherapy and radiation therapy. A systematic review. J. Gynecol. Obstet. Biol. Reprod..

[32] Höckel M. (2015). Long-term experience with (laterally) extended endopelvic resection (LEER) in relapsed pelvic malignancies. Curr. Oncol. Rep..

[33] Chou H.H., Wang C.C., Lai C.H., Hong J.H., Ng K.K., Chang T.C., Tseng C.J., Tsai C.S., Chang J.T. (2001). Isolated paraaortic lymph node recurrence after definitive irradiation for cervical carcinoma. Int. J. Radiat. Oncol. Biol. Phys..

[34] Singh A.K., Grigsby P.W., Rader J.S., Mutch D.G., Powell M.A. (2005). Cervix carcinoma, concurrent chemoradiotherapy, and salvage of isolated paraaortic lymph node recurrence. Int. J. Radiat. Oncol. Biol. Phys..

[35] Jeon W., Koh H.K., Kim H.J., Wu H.G., Kim J.H., Chung H.H. (2012). Salvage radiotherapy for lymph node recurrence after radical surgery in cervical cancer. J. Gynecol. Oncol..

[36] Kubota H., Tsujino K., Sulaiman N.S., Sekii S., Matsumoto Y., Ota Y., Soejima T., Yamaguchi S., Sasaki R. (2019). Comparison of salvage therapies for isolated para-aortic lymph node recurrence in patients with uterine cervical cancer after definitive treatment. Radiat. Oncol..

[37] Choi C.W., Cho C.K., Yoo S.Y., Kim M.S., Yang K.M., Yoo H.J., Seo Y.S., Kang J.K., Lee D.A., Lee K.H. (2009). Image-guided stereotactic body radiation therapy in patients with isolated para-aortic lymph node metastases from uterine cervical and corpus cancer. Int. J. Radiat. Oncol. Biol. Phys..

[38] Park H.J., Chang A.R., Seo Y., Cho C.K., Jang W.I., Kim M.S., Choi C. (2015). Stereotactic bodyradiotherapy for recurrent or oligometastatic uterinecervixcancer: A cooperative study of the Korean Radiation Oncology Group (KROG 14-11). Anticancer Res..

[39] Mendez L.C., Leung E., Cheung P., Barbera L. (2017). The role of stereotactic ablative bodyradiotherapy in gynaecological cancers: A systematic review. Clin. Oncol..

[40] Laliscia C., Fabrini M.G., Delishaj D., Morganti R., Greco C., Cantarella M., Tana R., Paiar F., Gadducci A. (2017). Clinical outcomes of stereotactic body radiotherapy in oligometastatic gynecological Cancer. Int. J. Gynecol. Cancer.

[41] Onal C., Gultekin M., Oymak E., Guler O.C., Yilmaz M.T., Yuce Sari S., Akkus Yildirim B., Yildiz F. (2020). Stereotactic radiotherapy in patients with oligometastatic or oligoprogressive gynecological malignancies: A multi-institutional analysis. Int. J. Gynecol. Cancer.

[42] Yamamoto K., Yoshikawa H., Shiromizu K., Saito T., Kuzuya K., Ryuichiro Tsunematsu R., Kamura T. (2004). Pulmonary metastasectomy for uterine cervical cancer: A multivariate analysis. Ann. Thorac. Surg..

[43] Mourton S.M., Sonoda Y., Abu-Rustum N.R., Bochner B.H., Barakat R.R., Chi D.S. (2007). Resection of recurrent cervical cancer after total pelvic exenteration. Int. J. Gynecol. Cancer.

[44] Moore D.H., Blessing J.A., McQuellon R.P., Thaler H.T., Cella D., Benda J., Miller D.S., Olt G., King S., Boggess J.F. (2004). Phase III study of cisplatin with or without paclitaxel in stage IVB, recurrent, or persistent squamous cell carcinoma of the cervix: A Gynecologic Oncology Group study. J. Clin. Oncol..

[45] Tewari K.S., Monk B.J. (2005). Gynecologic Oncology Group trials of chemotherapy for metastatic and recurrent cervical cancer. Curr. Oncol. Rep..

[46] Pfaendler K.S., Tewari K.S. (2016). Changing paradigms in the systemic treatment of advanced cervical cancer. Am. J. Obstet. Gynecol..

[47] Tewari K.S., Sill M.W., Penson R.T., Huang H., Ramondetta L.M., Landrum L.M., Oaknin A., Reid T.J., Leitao M.M., Michael H.E. (2017). Bevacizumab for advanced cervical cancer: Final overall survival and adverse event analysis of a randomised, controlled, open-label, phase 3 trial (Gynecologic Oncology Group 240). Lancet.

[48] Thigpen T., Shingleton H., Homesley H., Lagasse L., Blessing J. (1981). Cis-platinum in treatment of advanced or recurrent squamous cell carcinoma of the cervix: A phase II study of the Gynecologic Oncology Group. Cancer.

[49] Bonomi P., Blessing J.A., Stehman F.B., DiSaia P.J., Walton L., Major F.J. (1985). Randomized trial of three cisplatin dose schedules in squamous-cell carcinoma of the cervix: A Gynecologic Oncology Group study. J. Clin. Oncol..

[50] Thigpen J.T., Blessing J.A., Fowler W.C., Hatch K. (1986). Phase II trials of cisplatin and piperazinedione as single agents in the treatment of advanced or recurrent non-squamous cell carcinoma of the cervix: A Gynecologic Oncology Group Study. Cancer Treat. Rep..

[51] Lele S.B., Piver M.S. (1989). Weekly cisplatin induction chemotherapy in the treatment of recurrent cervical carcinoma. Gynecol. Oncol..

[52] Potter M.E., Hatch K.D., Potter M.Y., Shingleton H.M., Baker V.V. (1989). Factors affecting the response of recurrent squamous cell carcinoma of the cervix to cisplatin. Cancer.

[53] Thigpen T. (2003). The role of chemotherapy in the management of carcinoma of the cervix. Cancer J..

[54] Kumar L., Harish P., Malik P.S., Khurana S. (2018). Chemotherapy and targeted therapy in the management of cervical Cancer. Curr. Probl. Cancer.

[55] Arseneau J., Blessing J.A., Stehman F.B., McGehee R. (1986). A phase II study of carboplatin in advanced squamous cell carcinoma of the cervix (a Gynecologic Oncology Group Study). Investig. New Drugs.

[56] McGuire W.P., Arseneau J., Blessing J.A., DiSaia P.J., Hatch K.D., Given F.T., Teng N.N., Creasman W.T. (1989). A randomized comparative trial of carboplatin and iproplatin in advanced squamous carcinoma of the uterine cervix: A Gynecologic Oncology Group study. J. Clin. Oncol..

[57] Weiss G.R., Green S., Hannigan E.V., Boutselis J.G., Surwit E.A., Wallace D.L., Alberts D.S. (1990). A phase II trial of carboplatin for recurrent or metastatic squamous carcinoma of the uterine cervix: A Southwest Oncology Group study. Gynecol. Oncol..

[58] Meanwell C.A., Mould J.J., Blackledge G., Lawton F.G., Stuart N.S., Kavanagh J., Latief T.N., Spooner D., Chetiyawardana A.D. (1986). Phase II study of ifosfamide in cervical cancer. Cancer Treat. Rep..

[59] Sutton G.P., Blessing J.A., Adcock L., Webster K.D., DeEulis T. (1989). Phase II study of ifosfamide and mesna in patients with previously-treated carcinoma of the cervix: A Gynecologic Oncology Group study. Investig. New Drugs.

[60] Sutton G.P., Blessing J.A., DiSaia P.J., McGuire W.P. (1993). Phase II study of ifosfamide and mesna in nonsquamous carcinoma of the cervix: A Gynecologic Oncology Group study. Gynecol. Oncol..

[61] Morris M., Brader K.R., Levenback C., Burke T.W., Atkinson E.N., Scott W.R., Gershenson D.M. (1998). Phase II study of vinorelbine in advanced and recurrent squamous cell carcinoma of the cervix. J. Clin. Oncol..

[62] Lhommé C., Vermorken J.B., Mickiewicz E., Chevalier B., Alvarez A., Mendiola C., Pawinski A., Lentz M.A., Pecorelli S. (2000). Phase II trial of vinorelbine in patients with advanced and/or recurrent cervical carcinoma: An EORTC Gynaecological Cancer Cooperative Group study. Eur. J. Cancer.

[63] Muggia F.M., Blessing J.A., Waggoner S., Berek J.S., Monk B.J., Sorosky J., Pearl M.L. (2005). Evaluation of vinorelbine in persistent or recurrent nonsquamous carcinoma of the cervix: A Gynecologic Oncology Group Study. Gynecol. Oncol..

[64] Schilder R.J., Blessing J.A., Morgan M., Mangan C.E., Rader J.S. (2000). Evaluation of gemcitabine in patients with squamous cell carcinoma of the cervix: A phase II study of the gynecologic oncology group. Gynecol. Oncol..

[65] Schilder R.J., Blessing J., Cohn D.E. (2005). Evaluation of gemcitabine in previously treated patients with non-squamous cell carcinoma of the cervix: A phase II study of the Gynecologic Oncology Group. Gynecol. Oncol..

[66] McGuire W.P., Blessing J.A., Moore D., Lentz S.S., Photopulos G. (1996). Paclitaxel has moderate activity in squamous cervix cancer: A Gynecologic Oncology Group study. J. Clin. Oncol..

[67] Kudelka A.P., Winn R., Edwards C.L., Downey G., Greenberg H., Dakhil S.R., Freedman R.S., LoCoco S., Umbreit J., Delmore J.E. (1997). An update of a phase II study of paclitaxel in advanced or recurrent squamous cell cancer of the cervix. Anticancer Drugs.

[68] Curtin J.P., Blessing J.A., Webster K.D., Rose P.G., Mayer A.R., Fowler W.C., Malfetano J.H., Alvarez R.D. (2001). Paclitaxel, an active agent in nonsquamous carcinomas of the uterine cervix: A Gynecologic Oncology Group Study. J. Clin. Oncol..

[69] Garcia A.A., Blessing J.A., Vaccarello L., Roman L.D. (2007). Gynecologic Oncology Group Study: Phase II clinical trial of docetaxel in refractory squamous cell carcinoma of the cervix: A Gynecologic Oncology Group Study. Am. J. Clin. Oncol..

[70] Pearl M.L., Johnston C.M., McMeekin D.S. (2007). A phase II study of weekly docetaxel for patients with advanced or recurrent cancer of the cervix. Gynecol. Obstet. Investig..

[71] Bookman M.A., Blessing J.A., Hanjani P., Herzog T.J., Andersen W.A. (2000). Topotecan in squamous cell carcinoma of the cervix: A phase II study of the Gynecologic Oncology Group. Gynecol. Oncol..

[72] Muderspach L.I., Blessing J.A., Levenback C., Moore J.L. (2001). A Phase II study of topotecan in patients with squamous cell carcinoma of the cervix: A Gynecologic Oncology Group study. Gynecol. Oncol..

[73] Verschraegen C.F., Levy T., Kudelka A.P., Llerena E., Ende K., Freedman R.S., Edwards C.L., Hord M., Steger M., Kaplan A.L. (1997). Phase II study of irinotecan in prior chemotherapy-treated squamous cell carcinoma of the cervix. J. Clin. Oncol..

[74] Lhommé C., Fumoleau P., Fargeot P., Krakowski Y., Dieras V., Chauvergne J., Vennin P., Rebattu P., Roche H., Misset J.L. (1999). Results of a European Organization for Research and Treatment of Cancer/Early Clinical Studies Group phase II trial of first-line irinotecan in patients with advanced or recurrent squamous cell carcinoma of the cervix. J. Clin. Oncol..

[75] Goedhals L., van Wiyk A.L., Smith B.L., Fourie S.J. (2006). Pemetrexed (Alimta, LY231514) demonstrates clinical activity in chemonaive patients with cervical cancer in a phase II single-agent trial. Int. J. Gynecol. Cancer.

[76] Miller D.S., Blessing J.A., Bodurka D.C., Bonebrake A.J., Schorge J.O. (2008). Gynecologic Oncology Group: Evaluation of pemetrexed (Alimta, LY231514) as second line chemotherapy in persistent or recurrent carcinoma of the cervix: A phase II study of the Gynecologic Oncology Group. Gynecol. Oncol..

[77] Rose P.G., Blessing J.A., Arseneau J. (1996). Phase II evaluation of altretamine for advanced and recurrent squamous cell carcinoma of the cervix: A Gynecologic Oncology Group study. Gynecol. Oncol..

[78] Rose P.G., Blessing J.A., Morgan M., Van Le L., Waggoner S. (1998). Prolonged oral etoposide in recurrent or advanced squamous cell carcinoma of the cervix: A Gynecologic Oncology Group study. Gynecol. Oncol..

[79] Alberts D.S., Blessing J.A., Landrum L.M., Warshal D.P., Martin L.P., Rose S.L., Bonebrake A.J., Ramondetta L.M. (2012). Phase II trial of nab-paclitaxel in the treatment of recurrent or persistent advanced cervix cancer: A gynecologic oncology group study. Gynecol. Oncol..

[80] Micha J.P., Goldstein B.H., Birk C.L., Rettenmaier M.A., Brown J.V. (2006). Abraxane in the treatment of ovarian cancer: The absence of hypersensitivity reactions. Gynecol. Oncol..

[81] Bonomi P., Blessing J., Ball H., Hanjani P., DiSaia P.J. (1989). A phase II evaluation of cisplatin and 5-fluorouracil in patients with advanced squamous cell carcinoma of the cervix: A Gynecologic Oncology Group study. Gynecol. Oncol..

[82] Kaern J., Tropé C., Abeler V., Iversen T., Kjørstad K. (1990). A phase II study of 5-fluorouracil/cisplatinum in recurrent cervical cancer. Acta Oncol..

[83] Benjapibal M., Thirapakawong C., Leelaphatanadit C., Therasakvichya S., Inthasorn P. (2007). A pilot phase II study of capecitabine plus cisplatin in the treatment of recurrent carcinoma of the uterine cervix. Oncology.

[84] Daghestani N., Hakes T.B., Lynch G., Lewis J.L. (1983). Cervix carcinoma: Treatment with combination cisplatin and bleomycin. Gynecol. Oncol..

[85] Coleman R.E., Clarke J.M., Slevin M.L., Sweetenham J., Williams C.J., Blake P., Calman F., Wiltshaw E., Harper P.G. (1990). A phase II study of ifosfamide and cisplatin chemotherapy for metastatic or relapsed carcinoma of the cervix. Cancer Chemother. Pharmacol..

[86] Cervellino J.C., Araujo C.E., Sánchez O., Miles H., Nishihama A. (1995). Cisplatin and ifosfamide in patients with advanced squamous cell carcinoma of the uterine cervix. A phase II trial. Acta Oncol..

[87] Omura G.A., Blessing J.A., Vaccarello L., Berman M.L., Clarke-Pearson D.L., Mutch D.G., Anderson B. (1997). Randomized trial of cisplatin versus cisplatin plus mitolactol versus cisplatin plus ifosfamide in advanced squamous carcinoma of the cervix: A Gynecologic Oncology Group study. J. Clin. Oncol..

[88] Burnett A.F., Roman L.D., Garcia A.A., Muderspach L.I., Brader K.R., Morrow C.P. (2000). A phase II study of gemcitabine and cisplatin in patients with advanced, persistent, or recurrent squamous cell carcinoma of the cervix. Gynecol. Oncol..

[89] Rose P.G., Blessing J.A., Gershenson D.M., McGehee R. (1999). Paclitaxel and cisplatin as first-line therapy in recurrent or advanced squamous cell carcinoma of the cervix: A Gynecologic Oncology Group study. J. Clin. Oncol..

[90] Papadimitriou C.A., Sarris K., Moulopoulos L.A., Fountzilas G., Anagnostopoulos A., Voulgaris Z., Gika D., Giannakoulis N., Diakomanolis E., Dimopoulos M.A. (1999). Phase II trial of paclitaxel and cisplatin in metastatic and recurrent carcinoma of the uterine cervix. J. Clin. Oncol..

[91] Piver M.S., Ghamande S.A., Eltabbakh G.H., O’Neill-Coppola C. (1999). First-line chemotherapy with paclitaxel and platinum for advanced and recurrent cancer of the cervix—A phase II study. Gynecol. Oncol..

[92] Gebbia V., Caruso M., Testa A., Mauceri G., Borsellino N., Chiarenza M., Pizzardi N., Palmeri S. (2002). Vinorelbine and cisplatin for the treatment of recurrent and/or metastaticcarcinoma of the uterine cervix. Oncology.

[93] Smith H.O., Jiang C.S., Weiss G.R., Hallum A.V., Liu P.Y., Robinson W.R., Cheng P.C., Scudder S.A., Markman M., Alberts D.S. (2006). Tirapazamine plus cisplatin in advanced or recurrent carcinoma of theuterine cervix: A Southwest Oncology Group study. Int. J. Gynecol. Cancer.

[94] Long H.J., Bundy B.N., Grendys E.C., Benda J.A., McMeekin D.S., Sorosky J., Miller D.S., Eaton L.A., Fiorica J.V. (2005). Randomized phase III trial of cisplatin with or without topotecan in carcinoma of the uterine cervix: A Gynecologic Oncology Group Study. J. Clin. Oncol..

[95] Monk B.J., Sill M.W., McMeekin D.S., Cohn D.E., Ramondetta L.M., Boardman C.H., Benda J., Cella D. (2009). Phase III trial of four cisplatin-containing doublet combinations in stage IVB, recurrent, or persistent cervical carcinoma: A Gynecologic Oncology Group study. J.Clin. Oncol..

[96] Kitagawa Katsumata N., Shibata T., Kamura T., Kasamatsu T., Nakanishi T., Nishimura Ushijima K., Takano M., Satoh T., Yoshikawa H. (2015). Paclitaxel plus carboplatin versus paclitaxel plus cisplatin in metastatic or recurrent cervical cancer: The open-label randomized phase III trial JCOG0505. J. Clin. Oncol..

[97] Tewari K.S., Sill M.W., Long H.J., Penson R.T., Huang H., Ramondetta L.M., Landrum L.M., Oaknin A., Reid T.J., Leitao M.M. (2014). Improved survival with bevacizumab in advanced cervical cancer. N. Engl. J. Med..

[98] Bloss J.D., Blessing J.A., Behrens B.C., Mannel R.S., Rader J.S., Sood A.K., Markman M., Benda J. (2002). Randomized trial of cisplatin and ifosfamide with or without bleomycin in squamous carcinoma of the cervix: A Gynecologic Oncology Group study. J. Clin. Oncol..

[99] Zanetta G., Fei F., Parma G., Balestrino M., Lissoni A., Gabriele A., Mangioni C. (1999). Paclitaxel, ifosfamide and cisplatin (TIP) chemotherapy for recurrent or persistent squamous-cell cervical cancer. Ann. Oncol..

[100] Dimopoulos M.A., Papadimitriou C.A., Sarris K., Aravantinos G., Kalofonos C., Gika D., Gourgoulis G.M., Efstathiou E., Skarlos D., Bafaloukos D. (2002). Combination of ifosfamide, paclitaxel, and cisplatin for the treatment of metastatic and recurrent carcinoma of the uterine cervix: A phase II study of the Hellenic Cooperative Oncology Group. Gynecol. Oncol..

[101] Choi C.H., Kim T.J., Lee S.J., Lee J.W., Kim B.G., Lee J.H., Bae D.S. (2006). Salvage chemotherapy with a combination of paclitaxel, ifosfamide, and cisplatin for the patients with recurrent carcinoma of the uterine cervix. Int. J. Gynecol. Cancer.

[102] Van Luijk I.F., Coens C., van der Burg M.E., Kobierska A., Namer M., Lhomme C., Zola P., Zanetta G., Vermorken J.B., Gynecological Cancer Group of the European Organization for Research and Treatment of Cancer (2007). Phase II study of bleomycin, vindesine, mitomycin C and cisplatin (BEMP) in recurrent or disseminated squamous cell carcinoma of the uterine cervix. Ann. Oncol..

[103] Britten R.A., Liu D., Tessier A., Hutchison M.J., Murray D. (2000). ERCC1 expression as a molecular marker of cisplatin resistance in human cervical tumor cells. Int. J. Cancer.

[104] Mountzios G., Dimopoulos M.-A., Papadimitriou C. (2008). Excision repair cross-complementation group 1 enzyme as a molecular determinant of responsiveness to platinum-based chemotherapy for nonsmall-cell lung cancer. Biomark. Insights.

[105] Karageorgopoulou S., Kostakis I.D., Gazouli M., Markaki S., Papadimitriou M., Bournakis E., Dimopoulos M.A., Papadimitriou C.A. (2017). Prognostic and predictive factors in patients with metastatic or recurrent cervical cancer treated with platinum-based chemotherapy. BMC Cancer.

[106] Ryu H., Song I.C., Choi Y.S., Yun H.J., Jo D.Y., Kim J.M., Ko Y.B., Lee H.J. (2017). ERCC1 expression status predicts the response and survival of patients with metastatic or recurrent cervical cancer treated via platinum-based chemotherapy. Medicine.

[107] Rao D., Mallick A.B., Augustine T., Daroqui C., Jiffry J., Merla A., Chaudhary I., Seetharam R., Sood A., Gajavelli S. (2019). Excision repair cross-complementing group-1 (ERCC1) induction kinetics and polymorphism are markers of inferior outcome in patients with colorectal cancer treated with oxaliplatin. Oncotarget.

[108] Moore D.H., Tian C., Monk B.J., Long H.J., Omura G.A., Bloss J.D. (2010). Prognostic factors for response to cisplatin-based chemotherapy in advanced cervical carcinoma: A Gynecologic Oncology Group Study. Gynecol. Oncol..

[109] Monk B.J., Sill M.W., Burger R.A., Gray H.J., Buekers T.E., Roman L.D. (2009). Phase II trial of Bevacizumab in the treatment of persistent or recurrent squamous cell carcinoma of the cervix: A Gynecologic Oncology Group Study. J. Clin. Oncol..

[110] Bahadori H.R., Green M.R., Catapano C.V. (2001). Synergistic interaction between topotecan and microtubule-interfering agents. Cancer Chemother. Pharmacol..

[111] Tiersten A.D., Selleck M.J., Hershman D.L., Smith D., Resnik E.E., Troxel A.B., Brafman L.B., Shriberg L. (2004). Phase II study of topotecan and paclitaxel for recurrent, persistent, or metastatic cervical carcinoma. Gynecol. Oncol..

[112] Rosen V.M., Guerra I., McCormack M., Nogueira-Rodrigues A., Sasse A., Munk V.C., Shang A. (2017). Systematic review and network meta-analysis of bevacizumab plus first-line topotecan-paclitaxel or cisplatin-paclitaxel versus non-bevacizumab-containing therapies in persistent, recurrent, or metastatic cervical cancer. Int. J. Gynecol. Cancer.

[113] Lee N., Kim S.I., Lee M., Kim H.S., Kim J.W., Park N.H., Song Y.S. (2019). Bevacizumab efficacy and recurrence pattern of persistent and metastatic cervical cancer. In Vivo.

[114] Tinker A.V., Fiorino L., O’Dwyer H., Kumar A. (2018). Bevacizumab in metastatic, recurrent, or persistent cervicalcancer: The BC cancer experience. Int. J. Gynecol. Cancer.

[115] Kazuhiro Suzuki K., Shoji Nagao S., Takashi Shibutani T., Kasumi Yamamoto K., Tomoatsu Jimi T., Yano H., Kitai M., Shiozaki T., Matsuoka K., Yamaguchi S. (2019). Phase II trial of paclitaxel, carboplatin, and bevacizumab for advanced or recurrent cervical cancer. Gynecol. Oncol..

[116] Sturdza A., Hofmann S., Kranawetter M., Polterauer S., Grimm C., Krainer M., Kirisits C., Pötter R., Reinthaller A., Schwameis R. (2017). Increased genitourinary fistula rate after bevacizumab in recurrent cervicalcancer patients initially treated with definitive radiochemotherapy and image-guided adaptive brachytherapy. Strahlenther. Onkol..

[117] Iida T., Muramatsu T., Nakajima R., Narayama C., Narayama T., Goya K., Tsukada H., Maeda H., Machida T., Kuramoto T. (2019). Recurrent cervicalcancerwith intestinal perforation that was related to bevacizumab after long-term NSAIDs administration and was treated with laparoscopy-assisted anastomosis. Tokai J. Exp. Clin. Med..

[118] Tognarelli A., Faggioni L., Manassero F., Gadducci A., Selli C. (2019). A case report of endorectal displacement of a right ureteral stent following radiochemotherapy and bevacizumab. BMC Urol..

[119] Palavalli Parsons L.H., Roane B., Manders D.B., Richardson D.L., Kehoe S.M., Carlson M., Miller D.S., Lea J.S. (2018). Hypoalbuminemia is a predictive factor for fistula formation in recurrent cervical cancer. Am. J. Clin. Oncol..

[120] Tewari K.S., Sill M.W., Monk B.J., Penson R.T., Long H.J., Poveda A., Landrum L.M., Leitao M.M., Brown J., Reid T.J. (2015). Prospective validation of pooled prognostic factors in women with advanced cervical cancer treated with chemotherapy with/without bevacizumab: NRG Oncology/GOG Study. Clin. Cancer Res..

[121] Heeren A.M., Punt S., Bleeker M.C., Gaarenstroom K.N., van der Velden J., Kenter G.G., de Gruijl T.D., Jordanova E.S. (2016). Prognostic effect of different PD-L1 expression patterns in squamous cell carcinoma and adenocarcinoma of the cervix. Mod. Pathol..

[122] Naumann R.W., Hollebecque A., Meyer T., Devlin M.J., Oaknin A., Kerger J., López-Picazo J.M., Machiels J.P., Delord J.P., Evans T.R.J. (2019). Safety and efficacy of nivolumab monotherapy in recurrent or metastatic cervical, vaginal, or vulvar carcinoma: Results from the phase I/II CheckMate 358 trial. J. Clin. Oncol..

[123] Chung H.C., Ros W., Delord J.P., Perets R., Italiano A., Shapira-Frommer R., Manzuk L., Piha-Paul S.A., Xu L., Zeigenfuss S. (2019). Efficacy and safety of pembrolizumab in previously treated advanced cervical cancer: Results from the phase II KEYNOTE-158 study. J. Clin. Oncol..

[124] Cohen A.C., Roane B.M., Leath C.A. (2020). Novel therapeutics for recurrent cervical cancer: Moving towards personalized therapy. Drugs.

[125] Grau J.F., Farinas-Madrid L., Oaknin A. (2020). A randomized phase III trial of platinum chemotherapyplus paclitaxel with bevacizumab and atezolizumab versus platinum chemotherapy plus paclitaxel and bevacizumab inmetastatic(stage IVB), persistent, orrecurrentcarcinoma of thecervix: The BEATcc study (ENGOT-Cx10/GEICO 68-C/JGOG1084/GOG-3030). Int. J. Gynecol. Cancer.

[126] Wright A.A., Howitt B.E., Myers A.P., Dahlberg S.E., Palescandolo E., Van Hummelen P., MacConaill L.E., Shoni M., Wagle N., Jones R.T. (2013). Oncogenic mutations in cervical cancer: Genomic differences between adenocarcinomas and squamous cell carcinomas of the cervix. Cancer.

[127] McIntyre J.B., Wu J.S., Craighead P.S., Phan T., Kobel M., Lees-Miller S.P., Ghatage P., Magliocco A.M., Doll C.M. (2013). PIK3CA mutational status and overall survival in patients with cervical cancer treated with radical chemoradiotherapy. Gynecol. Oncol..

[128] Hou M.M., Liu X., Wheler J., Naing A., Hong D., Coleman R.L., Tsimberidou A., Janku F., Zinner R., Lu K. (2014). Targeted PI3K/AKT/mTOR therapy for metastatic carcinomas of the cervix: A phase I clinical experience. Oncotarget.

[129] Zammataro L., Lopez S., Bellone S., Pettinella F., Bonazzoli E., Perrone E., Zhao S., Menderes G., Altwerger G., Han C. (2019). Whole-exome sequencing of cervical carcinomas identifies activating ERBB2 and PIK3CA mutations as targets for combination therapy. Proc. Natl. Acad. Sci. USA.

[130] Liu J.J., Ho J.Y., Lee H.W., Baik M.W., Kim O., Choi Y.J., Hur S.Y. (2019). Inhibition of Phosphatidylinositol 3-kinase (PI3K) signaling synergistically potentiates antitumor efficacy of paclitaxel and overcomes paclitaxel-mediated resistance in cervicalcancer. Int. J. Mol. Sci..

[131] Tinker A.V., Ellard S., Welch S., Moens F., Allo G., Tsao M.S., Squire J., Tu D., Eisenhauer E.A., MacKay H. (2013). Phase II study of temsirolimus (CCI-779) in women with recurrent, unresectable, locally advanced or metastatic carcinoma of the cervix. A trial of the NCIC Clinical Trials Group (NCIC CTG IND 199). Gynecol. Oncol..

[132] Tang M., Liu Q., Zhou L., Chen L., Yang X., Yu J., Wang Y., Qiu H. (2019). The poly (ADP-ribose) polymerase inhibitor rucaparib suppresses proliferation and serves as an effective radiosensitizer in cervical cancer. Investig. New Drugs.

[133] Liu J.F., Gray K.P., Wright A.A., Campos S., Konstantinopoulos P.A., Peralta A., MacNeill K., Morrissey S., Whalen C., Dillon D. (2019). Results from a single arm, single stage phase II trial of trametinib and GSK2141795 in persistent or recurrent cervicalcancer. Gynecol. Oncol..

[134] Bianchi A., Lopez S., Altwerger G., Bellone S., Bonazzoli E., Zammataro L., Manzano A., Manara P., Perrone E., Zeybek B. (2019). PARP-1 activity (PAR) determines the sensitivity of cervical cancer to olaparib. Gynecol. Oncol..

[135] Cao P., Wang Y., Lv Y., Jiang N., Zhong L., Ma X., Xiao X., Ding D., Gu J., Lin L. (2019). PI3K p110α inhibition sensitizes cervical cancer cells with aberrant PI3K signaling activation to PARP inhibitor BMN673. Oncol. Rep..

[136] Roszik J., Ring K.L., Wani K.M., Lazar A.J., Yemelyanova A.V., Soliman P.T., Frumovitz M., Jazaeri A.A. (2018). Gene expression analysis identifies novel targets for cervical cancer therapy. Front. Immunol..

[137] Wallace N.A., Khanal S., Robinson K.L., Wendel S.O., Messer J.J., Galloway D.A. (2017). High-risk alphapapillomavirus oncogenes impair the homologous recombination pathway. J. Virol..

